# C‐type natriuretic peptide promotes human granulosa cell growth and estradiol production: Implications for early follicle development

**DOI:** 10.1002/rmb2.12626

**Published:** 2025-01-22

**Authors:** Yorino Sato, Kazuhiro Kawamura

**Affiliations:** ^1^ Department of Obstetrics and Gynecology Juntendo University Faculty of Medicine Bunkyo Tokyo Japan

**Keywords:** cGMP, CNP, granulosa cell, human, NPR2

## Abstract

**Purpose:**

To investigate the effects of C‐type natriuretic peptide (CNP) on human granulosa cell growth and elucidate its regulatory mechanisms.

**Methods:**

A human non‐luteinizing granulosa cell line (HGrC) developed from small antral follicles was used to assess the impact of CNP on cell proliferation and estrogen synthesis. cGMP production via the guanylate cyclase domain of the CNP receptor, natriuretic peptide receptor 2 (NPR2), was confirmed. The regulation of CNP encoding natriuretic peptide C (NPPC) and NPR2 by estradiol and oocyte‐derived factors (ODFs) was examined.

**Results:**

Besides detecting both NPPC and NPR2, CNP increased cellular proliferation. The specific action of CNP on cell proliferation was confirmed using siRNA transfection. CNP stimulated cGMP production, whereas a guanylate‐cyclase inhibitor suppressed CNP‐induced cell proliferation. Estradiol production was elevated by CNP treatment, accompanied by increased expression of estrogen synthetic enzymes. Furthermore, CNP upregulated NPR2 expression in cooperation with estradiol and ODFs, while estradiol increased NPPC expression.

**Conclusion:**

This study demonstrates CNP stimulation of human granulosa cell growth and suggests potential cross‐talk between these cells and oocytes. Further research on the simultaneous administration of CNP and estradiol may offer a promising approach for promoting early‐stage follicle development in infertility treatments for patients with poor ovarian reserve.

## Graphical abstract

This study demonstrated that CNP stimulates human granulosa cell proliferation and the potential for cross‐talk between these cells and oocytes. Further research on the simultaneous administration of CNP and E2 may offer a potential avenue for promoting early‐stage follicle development in infertility treatments for patients with poor ovarian reserve.

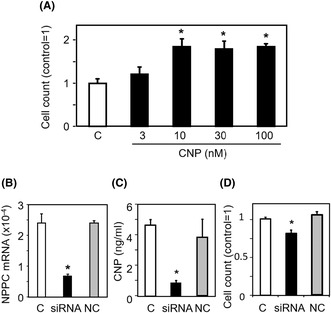

## 1 INTRODUCTION

Natriuretic peptides comprise three families: atrial natriuretic peptide (ANP), brain natriuretic peptide (BNP), and C‐natriuretic peptide (CNP). The ANP was initially discovered to be a diuretic peptide hormone,^1^, ^2^ while BNP and CNP were isolated from porcine brain extracts.^3^, ^4^ CNP is derived from the NPPC gene's precursor protein by cleavage into a 22‐amino acid residue peptide, but unlike ANP and BNP, it lacks direct natriuretic activity. Natriuretic peptide receptor B (NPR2), the cognate receptor for CNP, is a transmembrane protein localized to the cell membrane.^5^ It contains a guanylate cyclase domain that converts guanosine triphosphate (GTP) to 3′,5′‐cyclic GMP (cGMP) upon CNP binding to its extracellular domain. cGMP is degraded by cyclic nucleotide‐regulated phosphodiesterase (PDE), which regulates downstream cellular and physiological responses.^6^, ^7^, ^8^ The CNP expression has been reported in a wide variety of tissues, including endocrine tissues,^9^ the central nervous system,^10^ endothelial cells, and bone.^5^, ^11^, ^12^ The CNP derived from endothelial cells relaxed vascular smooth muscle via cGMP to exert anti‐hypertensive effects,^12^ whereas CNP produced by osteoblasts promotes cellular proliferation via an autocrine mechanism to facilitate bone formation.^13^, ^14^

Oocytes in large antral follicles spontaneously resume meiosis when extruded from the follicle, suggesting the presence of oocyte maturation inhibitor (OMI) within the follicle. Although the molecule remained unidentified for a long time, we previously demonstrated that CNP acted as the OMI.^15^ In response to the luteinizing hormone (LH) surge, which triggers oocyte maturation, phosphodiesterase 3A (PDE3A), an oocyte‐specific phosphodiesterase, degrades cAMP that had accumulated in oocytes to maintain meiotic arrest,^16^, ^17^ resulting in meiosis resumption.^18^ Before the LH surge, PDE3A activity is suppressed by cGMP produced in cumulus cells, which enters the oocyte via gap junctions to maintain meiotic arrest.^19^, ^20^ Spontaneous nuclear maturation observed in oocytes of large antral follicles in NPPC and NPR2 knockout mice before the LH surge^21^ prompted us to investigate CNP as a potential OMI. We demonstrated that granulosa cells produced CNP and secreted it into the follicular fluid, suppressing oocyte nuclear maturation via the CNP‐NPR2 signaling pathway.^15^ Furthermore, both CNP and cGMP levels declined after LH/human chorionic gonadotropin (hCG) stimulation, followed by meiosis resumption.^15^, ^20^ These results indicate that CNP is indeed the OMI. In contrast to the LH‐surge mediated decline in CNP production, NPR2 expression in cumulus‐oocyte complexes (COCs) is shown to be regulated by estradiol and oocyte‐derived factors (ODFs) such as GDF‐9, BMP15, and FGF8.^20^

In addition to suppressing oocyte maturation, we discovered that the CNP‐NPR2 signaling pathway stimulated the growth of early‐stage follicles. Using murine models, we found that CNP induced granulosa cell proliferations in secondary follicles, accompanied by increased follicular cGMP levels. We also demonstrated that NPPC and NPR2 expressions increased during follicle growth in mouse ovaries, and CNP treatment enhanced cGMP production in granulosa cells. Histological analysis revealed that CNP promoted the growth of primary and early secondary follicles to the late secondary stage in both in vitro and in vivo studies.^22^ Although follicle‐stimulating hormone (FSH) is currently used to stimulate antral follicle growth under controlled ovarian stimulation (COS) in reproductive medicine, no drug has been developed to promote the growth of early‐stage follicles. The development of such drugs is particularly important for patients with diminished ovarian reserve (DOR), as they could increase the number of FSH‐responsible antral follicles, potentially yielding a higher number of oocytes after COS. Therefore, in this study, we aimed to determine the effects of CNP on human granulosa cell proliferation and elucidate its regulatory mechanisms for the potential of CNP as an effective drug to treat infertile DOR patients.

## 2 MATERIALS AND METHODS

### 2.1 Cells

Given that in vivo treatment with CNP promoted the development of both preantral and antral follicles in mice,^22^ we used the human non‐luteinizing granulosa cell line (HGrC). This cell line was previously developed by obtaining granulosa cells from 3 to 5 mm diameter antral follicles of a 35‐year‐old woman and immortalized using lentivirus‐mediated gene introduction.^23^ We also employed two additional human granulosa cell types: KGN cells (a human ovarian granulosa‐like tumor cell line) provided by the RIKEN BRC through the MEXT's National BioResource Project,^24^ and SVOG cells (a immortalized human luteinizing granulosa cell line) kindly provided by Dr. Clara Salamanca from the University of British Columbia.^25^ All cell lines were cultured at 37°C in a 5% CO_2_ atmosphere using DMEM/F12 medium (Thermo Fisher Scientific, Waltham, MA, USA) supplemented with 10% fetal bovine serum (FBS, Biowest, Nuaillé, France) and Antibiotic‐Antimycotic (Thermo Fisher Scientific).

### 2.2 Animals and tissue samples

Female ICR mice were obtained from SLC Japan (Shizuoka, Japan) at 5–8 weeks of age. The mice were housed in a standard laboratory animal facility under controlled environment conditions (temperature: 22°C, humidity: 55%–65%, and light cycle: 12 h interval). All animal experiments were approved by the Animal Care and Use Committees at St. Marianna University School of Medicine and the International University of Health and Welfare School of Medicine. A human ovarian tissue sample was acquired from a company (Zyagen, CA).

### 2.3 Real‐time reverse transcription‐polymerase chain reaction (real‐time RT‐PCR)

Total RNA was extracted from three different types of ovarian granulosa cell lines using an RNeasy Micro kit (QIAGEN, Inc., CA) according to the manufacturer's protocol. Reverse transcription (RT) was performed using 1 μg of total RNA and a cDNA synthesis kit (Takara PrimeScript™ RT‐PCR Kit Perfect Real Time, Shiga, Japan). The primer sequences for b‐actin, FSH receptor (FSHR), LH receptor (LHR), NPPC, NPR2, and cytochrome P450 are shown in Table 1. Following the RT, real‐time PCR was conducted on a Light Cycler 96 (Roche Applied Science, IN). The PCR reaction began with a 10‐s incubation at 95°C, followed by 40 cycles of denaturation at 95°C for 5 s, and annealing and extension at 60°C for 30 s. Data were analyzed using the cycle threshold method to determine fold changes in expression. Transcript levels were normalized to b‐actin.

**TABLE 1:** Primer sequence.

b‐Actin	GTA TCC ATG AAA TAA GTG GTT ACA GG GCA GTA CAT AAT TTA CAC AGA AGC AAT
FSHR	TCT AAC AGG GTC TTC CTC TGC CTC AGT TCA ATG GCG TTC C
LHR	GAT GCA CAG TGG CAC CTT C CCT GCA ATT TGG TGG AAG AG
NPPC	GGT CTG GGA TGT TAG TGC AGC TA TAA AAG CCA CAT TGC GTT GGA
NPR2	GCT GAC CCG GCA AGT TCT GT ACA ATA CTC GGT GAC AAT GCA GAT
Cytochrome P450	ACC CTT CTG CGT CGT GTC GAA CTT CTA TGG CAT CTT TCA AAT CC

### 2.4 Cell counting

The effects of CNP on cell proliferation were studied using HGrC cells. Cells were seeded at a density of 1 × 10^3^ cells per well in 96‐well culture plate and preincubated overnight in serum‐free DMEM/F12 medium without phenol red (Thermo Fisher Scientific). The cells were then treated with or without different doses of CNP (Merck, Darmstadt, Germany) for 48 h in the serum‐free medium. Cell viability was assessed using the trypan blue exclusion test (Thermo Fisher Scientific). Viable cells were counted using a hemocytometer (NanoEnTek Inc. Seoul, Korea), based on their ability to exclude the trypan blue.

### 2.5 siRNA transfection for suppression of endogenous CNP expression

The siRNAs were designed and purchased from the riboxx RNA Technologies (Meiβner Str., Germany). The CNP‐specific siRNA sequence was 5′‐UCA CCU GUC CUG UGC GAA A‐3′, whereas the negative control siRNA (iBONi siRNA negative control) was 5′‐AGG UAG UGU AAU CGC CUU G‐3′. Cells were transfected with siRNAs using a Lipofectamine 2000 (Thermo Fisher Scientific) according to the manufacturer's standard protocol. Briefly, HGrC cells were grown to 80% confluence and then transfected with 100 nM CNP siRNA. The cells were cultured for an additional 48 h to measure NPPC mRNA levels, confirming the suppression of CNP expression. Cells with or without siRNA transfection were cultured for 72 h, and the culture media were subjected to ELISA to determine CNP protein levels after CNP siRNA treatment. CNP levels were measured using an ELISA kit with a primary antibody against the 22 amino acid residues of the whole CNP peptide (Cloud‐Clone Corp, Katy, TX, USA). Absorbance was measured at 450 nm using an iMark microplate reader (BioRad, Hercules, CA, USA).

### 2.6 Measurement of cGMP levels in HGrC cells

HGrC cells were pre‐cultured overnight in DMEM/F12 medium supplemented with 10% FBS. The cells were then seeded in 96‐well culture plates at a density of 5 × 10^4^ cells per well. In serum‐free medium, cells were treated for 15 min with 30 nM CNP, with or without zaprinast (Merck), a PDE5 inhibitor. Following treatment, cGMP concentrations in the cell culture medium were assessed using an ELISA kit (Cayman Chemical, Ann Arbor, MI, USA) according to the manufacturer's instructions. To explore the role of CNP‐mediated cGMP production in the HGrC cell proliferation, cells were treated with 30 nM CNP and different doses of LY83583 (Merck), a guanylate cyclase inhibitor. Cell counting was then conducted as previously described. The CNP dose used in this experiment was determined based on the results of the cell proliferation test evaluating the impact of CNP.

### 2.7 Measurement of estradiol and cytochrome P450 levels in HGrC cells

HGrC cells were seeded in 6‐well plates at a density of 5 × 10^4^ cells per well and pre‐cultured overnight in DMEM/F12 medium with 10% FBS. The culture medium was then supplemented with 10 μM androstenedione (4‐androstene‐3,17‐dione; Tokyo Chemical Industry Co., Ltd., Tokyo, Japan) as a substrate for estradiol. Cells were treated with different doses of CNP or 5 mM forskolin in serum‐free medium. At 48 h after treatment, estradiol levels in the cell culture medium were determined using an ELISA kit (Cloud‐Clone) according to the manufacturer's protocol. To measure changes in transcript levels of the estrogen synthetic enzyme cytochrome P450 *following CNP treatment*, HGrC cells were seeded in 96‐well culture plates at a density of 5 × 10^4^ cells per well and pre‐cultured overnight in DMEM/F12 medium supplemented with 10% FBS. Cells were then treated with different doses of CNP or 5 μM forskolin in the serum‐free medium for 48 h. Real‐time RT‐PCR, as described previously, was conducted to measure the transcript levels of cytochrome P450 in the cells.

### 2.8 Effects of oocyte co‐culture and oocyte‐derived factors on NPPC and NPR2 expressions in HGrC cells

HGrC cells were seeded in 96‐well culture plate at a density of 5 × 10^4^ cells per well and pre‐cultured overnight in DMEM/F12 medium with serum. To prepare oocytes for co‐culture, mice were treated with 5 IU equine chorionic gonadotropin (eCG) followed by 5 IU human chorionic gonadotropin (hCG) (ASKA Animal Health Co., Ltd., Tokyo, Japan) to induce ovulation. Cumulus‐oocyte complexes (COCs) were collected from oviducts at 14 h after hCG administration. Denuded oocytes for co‐culture were prepared by mechanical removing cumulus cells from COCs in the presence of hyaluronidase (Merck). HGrC cells were then co‐cultured with 50 denuded oocytes per well in serum‐free medium, which was supplemented with or without ODFs (500 ng/mL mouse GDF9 [R&D Systems, Minneapolis, MN, USA], human BMP15 [R&D Systems], and human FGF8 [BioVision Inc., Milpitas, MO, USA]), 100 nM E2 (Merck), and 30 nM CNP in diverse combinations. The concentration of ODFs to be supplemented with the culture medium was determined based on a previous study.^20^ At 48 h after culture, NPPC and NPR2 transcript levels were measured using quantitative RT‐PCR as previously described.

### 2.9 Statistical analysis

Statistical analysis was conducted using Prism software (GraphPad Software Inc., San Diego, CA, USA). One‐way ANOVA was used to test the statistical significance, followed by Dunnett's multiple comparisons post‐test. Data are presented as mean ± standard error of the mean (SEM). A *p*‐value less than 0.05 was considered statistically significant.

## 3 RESULTS

### 3.1 Expression of NPPC, NPR2, and gonadotropin receptors in diverse granulosa cell lines

**FIGURE 1:**
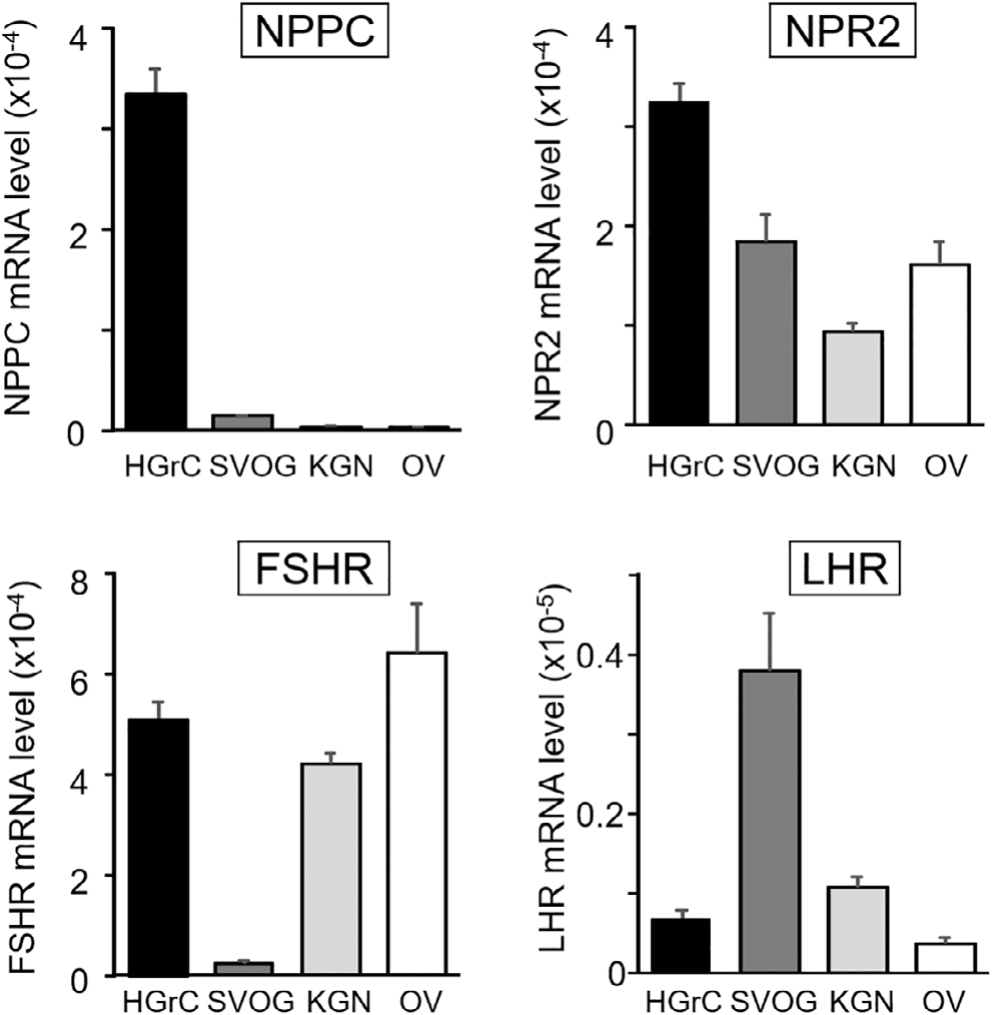
Expression of NPPC, NPR2, and gonadotropins receptors in diverse granulosa cell lines. To identify suitable cell lines for studying the roles of CNP‐NPR2 signaling in human granulosa cells, we examined the expression of NPPC (CNP precursor protein; cleaved into CNP protein), NPR2 receptor (the cognate receptor for CNP), FSHR, and LHR in three different human granulosa cell lines: HGrC, SVOG, and KGN. The transcript levels of these genes were quantified by real‐time RT–PCR and normalized to an internal control (b‐actin) (mean ± SEM, *n* = 6). FSHR, follicle‐stimulating hormone receptor; HGrC, human non‐luteinizing granulosa cell line; KGN, human ovarian granulosa‐like tumor cell line; LHR, luteinizing hormone receptor; NPPC, natriuretic peptide precursor type C; NPR2, natriuretic peptide receptor 2; OV, human ovary cDNA; SVOG, human luteinizing granulosa cell line.

To identify suitable cell lines for studying the roles of CNP‐NPR2 signaling in human granulosa cell growth, we examined the expression of NPPC, NPR2, and gonadotropin receptors in three different human granulosa cell lines (HGrC, SVOG, and KGN), using human ovary cDNA as a control. As shown in Figure 1, NPPC transcript levels were high in HGrC cells but low in other cell lines. In contrast, NPR2 receptor expression was detected in all cell lines with no significant differences in expression levels. Furthermore, HGrC cells expressed FSHR and LHR at levels similar to those observed in human ovarian samples. Based on these data, HGrC cells were selected as an in vitro model to determine the roles of CNP in human ovarian follicle growth.

### 3.2 CNP‐mediated stimulation of cell proliferation

**FIGURE 2:**
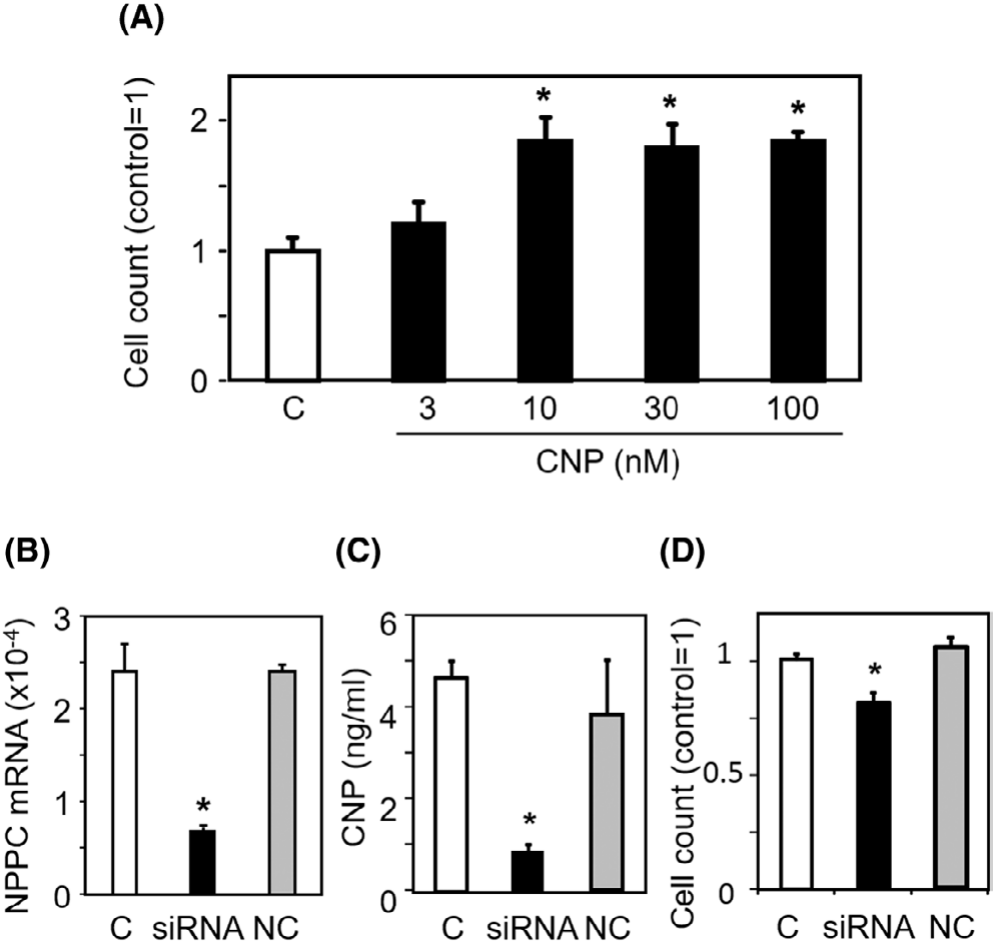
CNP‐mediated stimulation of cell proliferation. To assess the effect of CNP on human granulosa cell proliferation, HGrC cells were cultured with 3–100 nM CNP. The specific action of CNP on cell proliferation was confirmed by suppressing CNP expression using siRNA transfection. (A) Dose‐dependent effect of CNP on HGrC cell proliferation. At 48 h of culture, viable cells was quantified using a trypan blue exclusion assay (mean ± SEM, *n* = 6). The ratio was calculated with control set to 1. *p < 0.05, versus control (C). (B) Suppression of NPPC mRNA levels by CNP siRNA treatment. HGrC cells were transfected with siRNAs targeting CNP and a negative control. Cells were cultured for an additional 48 h before measuring NPPC mRNA levels (mean ± SEM, *n* = 4). (C) Suppression of CNP levels by CNP siRNA treatment. At 72 h after siRNAs transfection, CNP concentration in the culture medium supernatant was quantified by ELISA (mean ± SEM, *n* = 6). (D) CNP siRNA inhibition of HGrC cell proliferation. At 48 h after siRNAs transfection, viable cells were quantified using a trypan blue exclusion assay (mean ± SEM, *n* = 6). The ratio was calculated with control set to 1. *p < 0.05, versus control (C). C, control without siRNA transfection; NC, control with negative control siRNA transfection.

To assess the effect of CNP on human granulosa cell proliferation, HGrC cells were cultured with different concentrations of CNP. CNP treatment significantly increased cellular proliferation in a dose‐dependent manner (Figure 2A, *p* < 0.05). The specific action of CNP on cell proliferation was confirmed by suppressing CNP expression using siRNA transfection. CNP siRNA treatment significantly reduced cellular NPPC mRNA levels and CNP protein levels in the culture medium (Figure 2B,C, *p* < 0.05, respectively), resulting in suppression of HGrC cells proliferation (Figure 2D, *p* < 0.05, vs. control).

### 3.3 Involvement of cGMP production in CNP‐induced cell proliferation

**FIGURE 3:**
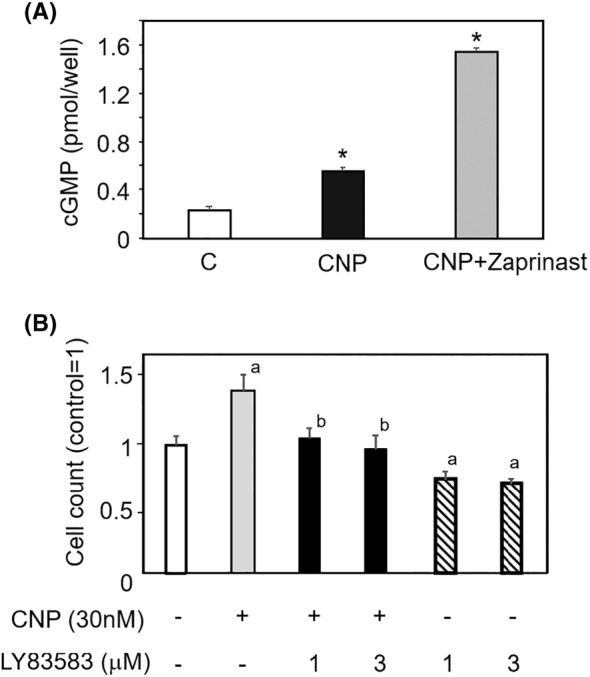
Involvement of cGMP production in CNP‐induced cell proliferation. To evaluate the impact of CNP on cGMP production in HGrC cells, the cells were treated with 30 nM CNP, and zaprinast, a PDE5 inhibitor that prevented cGMP degradation. The role of guanylate cyclase in CNP‐induced HGrC cell proliferation was further investigated using LY83583, a guanylate cyclase inhibitor. (A) Increase in cGMP production by CNP treatment. At 15 min after culture, cGMP concentrations in the culture medium supernatant were quantified using an ELISA (mean ± SEM, *n* = 6) and expressed as pmol/well. *p < 0.05, versus control (C). (B) Suppression of CNP‐induced HGrC cell proliferation by LY83583 treatment. At 48 h after culture, viable cells were quantified using a trypan blue exclusion assay (mean ± SEM, *n* = 6). The ratio was calculated with control set to 1. *p < 0.05, versus control. ‘a’ is compared to the control, and ‘b’ is compared to CNP 30nM, and the former has a significant difference compared to the latter.

CNP‐induced granulosa cell proliferation in mice has been shown to be mediated by cGMP production following the binding of CNP to the NPR2 receptor.^22^ To evaluate the impact of CNP on cGMP production in HGrC cells, we treated the cells with CNP, and zaprinast (ZP), a PDE5 inhibitor that prevents cGMP degradation in murine granulosa cells.^22^ At 15 min after treatment, cGMP concentrations in the cell culture medium were higher in the CNP alone and both CNP and ZP groups compared to the control group. However, the increase in cGMP levels was more pronounced in the ZP group (Figure 3A).

We further investigated the role of guanylate cyclase in CNP‐induced HGrC cell proliferation using LY83583, a guanylate cyclase inhibitor that blocks the conversion of GTP to cGMP. Although 30 nM CNP treatment significantly increased in cell number (*p* < 0.05), co‐treatment with CNP and 1 or 3 μM LY83583 suppressed CNP‐induced cell proliferation (Figure 3B). LY83583 alone inhibited cell proliferation, presumably by blocking the effect of endogenous CNP acting on the cells in an autocrine manner (Figure 3B, *p* < 0.05).

### 3.4 CNP stimulation of estrogen production

**FIGURE 4:**
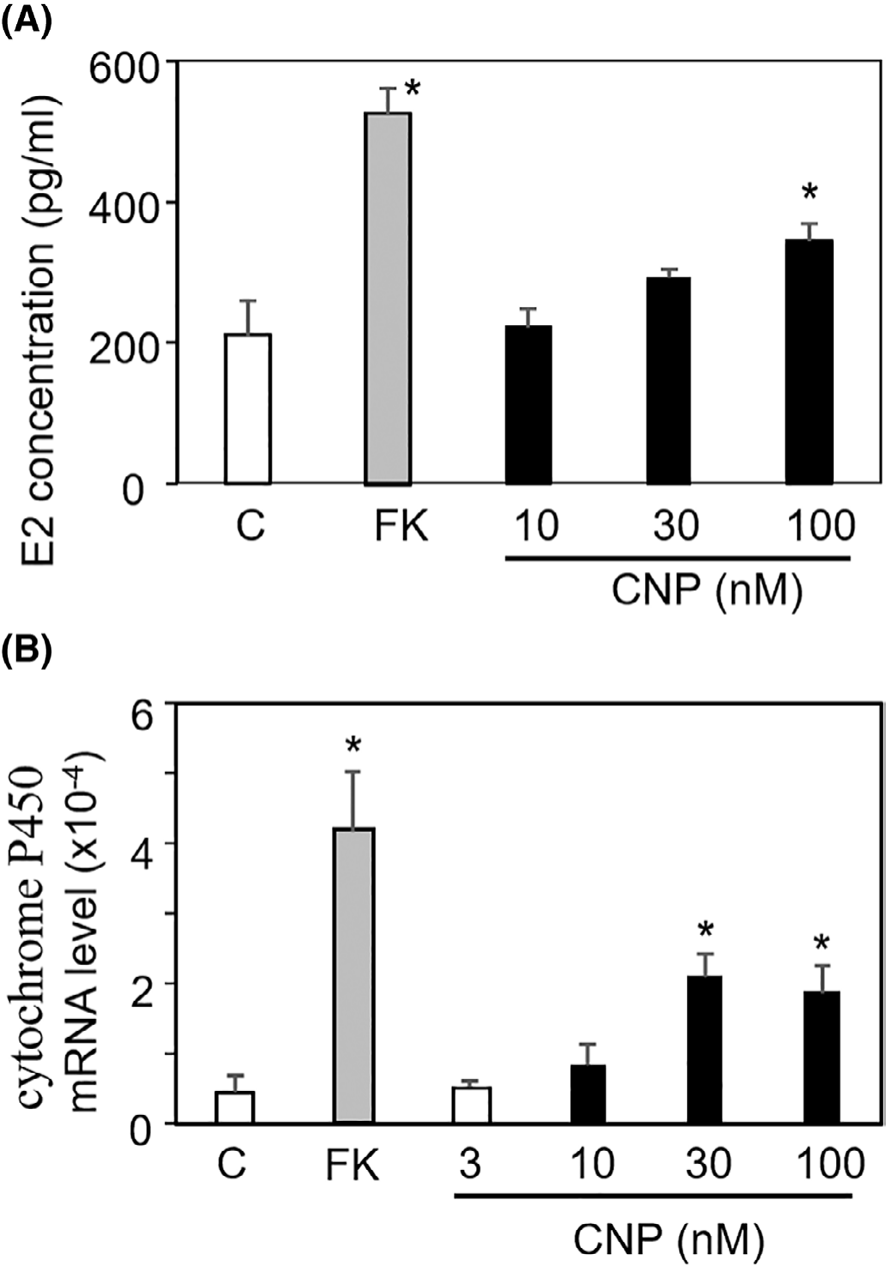
CNP stimulation of estrogen production. To assess the effect of CNP on cellular function in human granulosa cell growth, HGrC cells were cultured with 10–100 nM CNP in the presence of 10 μM androstenedione as a substrate for estradiol. As a positive control, treatment with 5 μM forskolin (an adenylyl cyclase stimulator) was applied. (A) Enhanced estradiol production by CNP treatment. At 48 h after culture, the concentration of estradiol in the culture medium supernatant was quantified by ELISA (mean ± SEM, *n* = 6). *p < 0.05, versus control (C). FK, forskolin. (B) Increase in the estrogen synthetic enzyme, cytochrome P450 expression by CNP treatment. At 48 h after culture, total RNA was extracted from the cells and cytochrome P450 transcript levels were quantified by real‐time RT–PCR and normalized to an internal control (b‐actin) (mean ± SEM, *n* = 6). *p < 0.05, versus control (C).

To gain further insights into the roles of CNP in human granulosa cell growth, we measured estrogen production from the cells to assess cellular function. Forskolin, an adenylyl cyclase stimulator, was used as a positive control. E2 production was elevated by CNP treatment in a concentration‐dependent manner, with a significant increase observed at 100 nM (Figure 4A, *p* < 0.05). Furthermore, significant increases in the estrogen synthetic enzyme, cytochrome P450, were observed with 30 and 100 nM CNP treatment (Figure 4B, *p* < 0.05).

### 3.5 Regulation of NPR2 and CNP expressions by E2 and ODFs

Previous research has indicated cross‐talk between oocytes and granulosa cells in the upregulation of NPR2 expression.^21^ Both ODFs and estradiol treatments have been demonstrated to increase NPR2 expression in murine granulosa cells.^20^, ^21^ To investigate the roles of this cross‐talk in regulating cell proliferation in HGrC cells, we determined changes in NPR2 and NPPC expressions, as well as cell proliferation, by co‐treating HGrC cells with oocytes. The cells were also exposed to estradiol, CNP, and estradiol + CNP in the presence or absence of ODFs.

**FIGURE 5:**
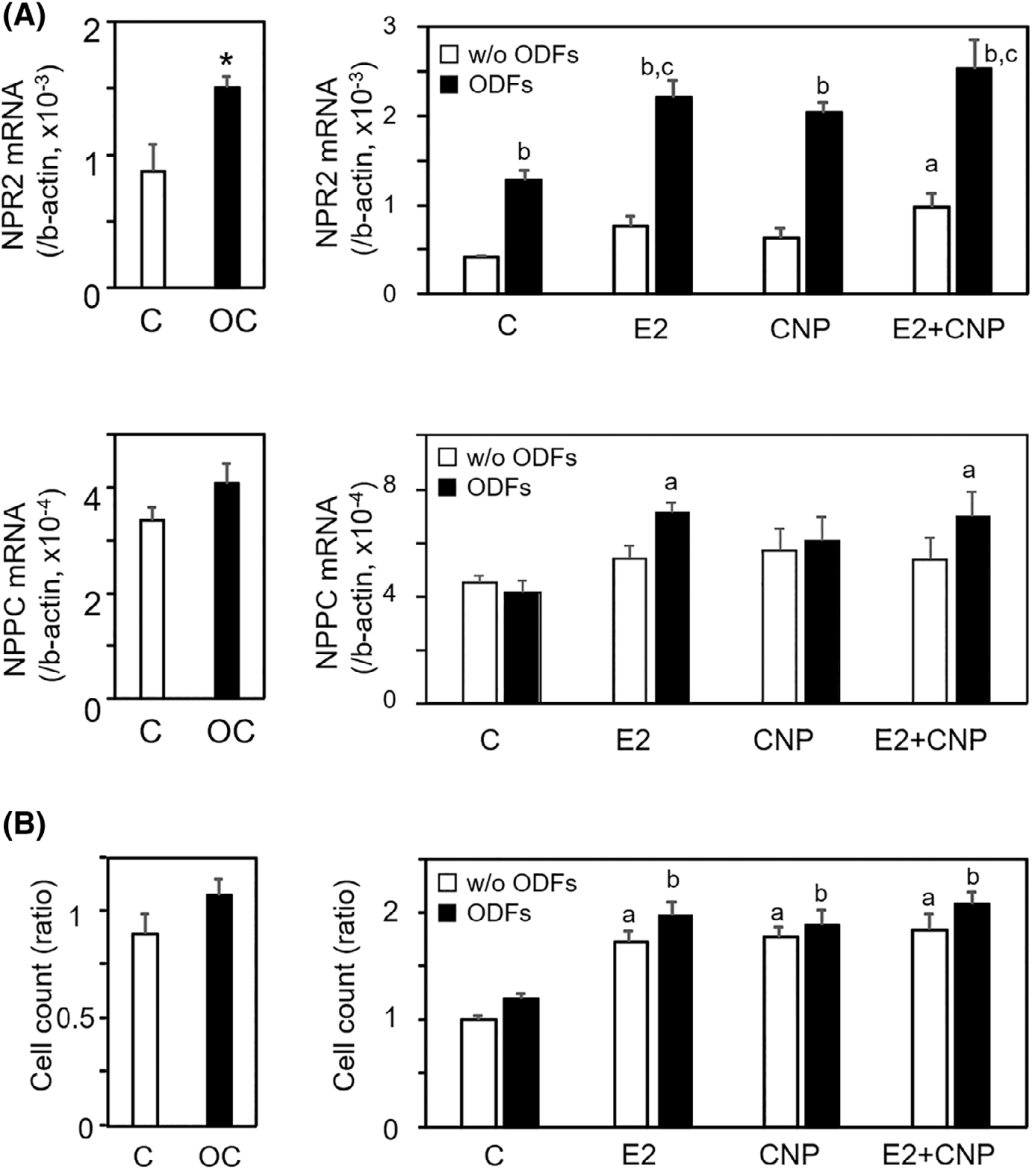
Regulation of NPR2 and CNP expressions by E2 and ODFs. To investigate the roles of a cross‐talk between oocytes and granulosa cells in regulating cell proliferation in HGrC cells, we evaluated changes in NPR2 and NPPC expressions, as well as cell proliferation, through co‐treatment of HGrC cells with fifty denuded oocytes (OC) per well. The cells were treated with 100 nM estradiol (E2), 30 nM CNP, and a combination of 100 nM estradiol (E2) + 30 nM CNP in the presence or absence of oocyte‐derived factors (ODFs), which included 500 ng/mL GDF9, BMP15, and FGF8. (A) Effect on NPR2 and NPPC expressions. At 48 h after culture, total RNA was extracted from the cells and the transcript levels of NPR2 and NPPC were quantified by real‐time RT–PCR, normalized to an internal control (b‐actin) (mean ± SEM, *n* = 6). In the upper left panel: *p < 0.05; right panel: a; versus control without ODFs, b; versus without ODFs, c; versus control with ODFs. In the lower right panel: a; versus control with ODFs. Letter symbols indicate significant differences (*p* < 0.05). (B) Effect on cell proliferation. At 48 h of culture, viable cells were quantified using a trypan blue exclusion assay (mean ± SEM, *n* = 6). The ratio was calculated with control set to 1: a; versus control without ODFs, b; versus with ODFs. Letter symbols indicate significant differences (*p* < 0.05).

As shown in Figure 5A, both ODFs treatment and the co‐culture of HGrC cells with oocytes increased NPR2 transcript levels (*p* < 0.05). Although the increase in NPR2 expression was not statistically significant with estradiol treatment without ODFs, NPR2 levels increased with co‐treatment of estradiol and CNP, suggesting a synergistic effect (Figure 5A, *p* < 0.05). In the presence of ODFs, treatment with estradiol, CNP, and estradiol + CNP increased NPR2 transcript levels (Figure 5A, *p* < 0.05), whereas no synergistic effect was found in the estradiol + CNP group (Figure 5A). In contrast to NPR2 expression, neither ODFs treatment nor co‐culture of HGrC cells with oocytes stimulated NPPC expression (Figure 5A). In the presence of ODFs, estradiol treatment increased in the NPPC expression (Figure 5B, *p* < 0.05), whereas no synergistic effect was found in the estradiol + CNP group (Figure 5B).

Regarding cell proliferation, co‐culture of HGrC cells with oocytes showed no stimulatory effect (Figure 5B). Although treatment of HGrC cells with estradiol, CNP, and estradiol + CNP induced cell proliferation (Figure 5B, *p* < 0.05), ODFs did not exhibit a stimulatory effect on cell proliferation in any treatment groups (Figure 5B).

## 4 DISCUSSION

In patients with POI and DOR, increasing the number of antral follicles by stimulating the development of FSH‐independent early‐stage follicles is effective in retrieving more oocytes during COS.^15^, ^26^ However, no pharmaceutical agents have been developed to induce early‐stage follicle development. To identify potential agents for this purpose, we employed temporal gene expression analysis during follicle development using DNA microarrays of mouse ovaries, collecting ovarian samples at different timepoints under gonadotropin treatments.^27^ This approach enabled us to identify factors whose expression increases after FSH administration and, in the presence of their receptors in the follicles, promote early follicle development through autocrine action. Our studies led us to identify CNP as a potential candidate and demonstrated that CNP stimulated the development of early secondary follicles to the late secondary stage in an FSH‐independent manner, as evidenced by both in vitro and in vivo murine studies.^22^ In this study, we conducted in vitro tests on non‐luteinized human granulosa cell lines to obtain preclinical proof of concept (POC), facilitating the transition from our laboratory findings in animals to potential clinical applications. Our results demonstrated that CNPs enhanced cellular proliferation via an increase in cGMP and the cellular steroidogenic potential. While previous studies have identified estradiol and ODFs as local regulators of CNP expression in the ovary,^20^, ^21^ our findings indicate that CNP upregulated NPR2 expression in non‐luteinized human granulosa cells in cooperation with estradiol and ODFs. These findings suggest the importance of cross‐talk between human granulosa cells and oocytes for inducing early follicle development via the CNP‐NPR2 signaling pathway. This insight may lead to novel therapeutic approaches for patients with POI and DOR, potentially improving outcomes in assisted reproductive technologies.

Given the intricate signaling pathways regulating early‐stage follicle development, which comprise both oocytes and surrounding somatic cells,^28^ follicle or organ cultures represent an optimal approach for evaluating the impact of CNP on follicle growth. Ideally, such studies would use ovaries with normal ovarian reserve must be obtained from young women. However, procuring normal ovaries from young women is ethically challenging. Therefore, we used human granulosa cell lines to obtain preclinical POC. Because follicles develop until ovulation by proliferating granulosa cells in a non‐luteinized state,^29^ studies examining the effects of CNP on early‐stage follicle development are suitable for using a granulosa cell line in a non‐luteinization state. We evaluated three different human granulosa cell lines: the non‐luteinizing granulosa cell line (HGrC),^23^ the luteinizing granulosa cell line (SVOG),^30^ and the granulosa cell tumor cell line (KGN).^24^ Among these cell lines, we confirmed that the HGrC cells expressed high levels of NPPC and its cognate receptor, NPR2, making them the most suitable for our study.

Our previous study demonstrated CNP promotion of early‐stage follicle development in mice via cGMP production.^22^ In the present study, we found that CNP treatment stimulated intracellular cGMP production and subsequent cell proliferation in the HGrC cells, suggesting the potential of CNP for stimulating early follicle growth in humans. A previous study using a serum‐free culture system of rat ovarian follicles demonstrated that cGMP analogs inhibited apoptosis and increased cellular viability in granulosa cells, leading to enlargement of the follicular diameter.^31^ However, the precise mechanism by which CNP induces the proliferation of granulosa cells via cGMP production remains unclear. As cGMP has been shown to activate cGMP‐dependent kinases (PKGs), which are involved in cell proliferation and promote cell growth in other cell types, such as bone marrow stromal cells,^32^ human kidney cancer cells,^33^ and vascular smooth muscle cells,^34^ it is plausible that granulosa cells may proliferate through a similar mechanism.

Estradiol, a hormone produced in granulosa cells, has been demonstrated to serve as an indicator of cell viability in a number of studies.^35^, ^36^, ^37^ Estradiol also plays a pivotal role in the development of early‐stage follicles by promoting granulosa cell proliferation.^38^ Although serum estradiol levels correlate with follicular diameter, oocytes associated with low serum estradiol levels relative to follicular size exhibit reduced rates of fertilization, embryo development, and pregnancy.^39^ In this study, we found that CNP treatment resulted in elevated expression of the estradiol synthase cytochrome P450 in HGrC cells and augmented estradiol levels in the culture supernatant. While it cannot be excluded that the elevated estradiol levels reflect an increase in cell number induced by CNP treatment, the cytochrome P450 transcript levels were quantified as expression per cell with β‐actin serving as an internal control. This suggests that CNP promoted estrogen synthesis via increased cytochrome P450 expression, rather than solely through increased cell numbers.

In our previous study on the changes in ovarian expression of CNP during the estrus cycle, we employed DNA microarray analyses to determine the temporal gene expression profiles in mouse ovaries. Our findings indicated that CNP expression increased following FSH administration, and that elevated CNP levels rapidly declined upon LH stimulation. Moreover, CNP levels in human follicular fluid also declined rapidly following LH stimulation, indicating that CNP production is regulated by an endocrine pathway mediated by gonadotropins.^15^ Furthermore, our previous findings indicate that the expression of NPR2 was low in small follicles and increased as the follicle developed.^22^ Synthetic estrogen diethylstilbestrol (DES) has been demonstrated to elevate NPR2 mRNA levels and stimulate CNP production in cumulus cells derived from mouse COCs.^20^ Additionally, rat granulosa cells obtained after in vivo administration of DES showed increasing NPR2 mRNA levels and subsequent cGMP production.^40^ Given that endogenous estrogen is synthesized de novo in granulosa cells within ovarian follicles, these findings provide compelling evidence for a regulatory mechanism controlling NPR2 expression by estrogen within the ovary.

In contrast to a previous study on rats,^40^ our current study demonstrated that treatment with 17β‐estradiol alone did not stimulate NPR2 expression in HGrC cells. This discrepancy may be attributed to the distinct estrogens used. While DES was employed in the rat experiments, we utilized 17β‐estradiol, the most abundant endogenous estrogen, to better mimic the in vivo environment. 17β‐estradiol has a lower binding affinity for the estrogen receptors (ERa and ERb) than DES,^41^ which may explain why 17β‐estradiol alone did not stimulate NPR2 expression in our study.

In addition to estrogen, ODFs have been demonstrated to regulate the local expression of NPR2 in murine cumulus cells in the ovary.^21^ ODFs comprise diverse growth factors secreted by oocytes that impact the growth and function of surrounding somatic cells.^42^ Among ODFs, the transforming growth factor (TGF)‐β superfamily^43^, ^44^, ^45^ and the fibroblast growth factor (FGF) family^46^, ^47^, ^48^ have been extensively studied. In particular, GDF9, BMP15, and FGF 8 are produced by oocytes in both dormant primordial follicles and developing follicles, serving as local regulators of follicle development in the ovary.^47^, ^49^ In our study, co‐culturing HGrC cells with mouse oocytes increased NPR2 transcript levels, suggesting that the mouse ODFs could contribute to NPR2 upregulation in human cells. This effect may be explained by the high amino acid sequence identity between mouse and human GDF‐9 (90%), BMP15 (70%), and FGF8 (100%).^44^, ^45^, ^50^, ^51^ Although previous studies have demonstrated that ODFs stimulate granulosa cell proliferations,^52^, ^53^, ^54^ our results did not show a significant effect of ODFs on cell proliferation. These discrepancies may be attributed to differences in cell types, and further research is necessary to elucidate the underlying causes.

Our findings demonstrated that CNP stimulated NPR2 expression in HGrC cells, consistent with previous reports indicating that CNP upregulates NPR2 expressions in other cell types.^55^, ^56^ Furthermore, we found a synergistic effect between 17β‐estradiol and CNP on stimulating NPR2 expression. However, no additional synergy was identified with the further addition of ODFs. It can be postulated that NPR2 expression reached a plateau with the combination of 17β‐estradiol and CNP treatment, making it challenging to examine the combined effect of all three substances in this system. Consistent with previous studies reporting increased granulosa cell proliferation in response to estradiol,^38^, ^57^, ^58^ our results demonstrated that estradiol stimulation induced cell proliferation in HGrC cells. Although CNP treatment also stimulated granulosa cell proliferation, we found no evidence of a synergistic effect when estradiol and CNP were used in combination. This lack of synergistic action may be attributed to the cells reaching a plateau of proliferation following either 17β‐estradiol or CNP treatment.

While our previous study demonstrated the endocrine regulation of CNP expression by gonadotropins,^15^ the local regulation of CNP expression in the ovary remained unclear. The present study found a significant increase in CNP expression following 17β‐estradiol treatment when administered with ODFs in HGrC cells. These results are inconsistent with a previous report demonstrating that 17β‐estradiol alone increases CNP mRNA expression in granulosa cells isolated from mouse follicles.^59^ This discrepancy may be explained by species differences between mouse and human granulosa cells.

Despite previous attempts at in vitro maturation (IVM) of oocytes to improve the clinical outcome of IVF, primarily in patients with polycystic ovary syndrome,^60^ IVM has resulted in lower birth rates compared to standard COS.^61^, ^62^ A recently reported method incorporating a pre‐maturation or capacitation step for IVM is the CAPA‐IVM approach.^63^ This method applies our original concept to synchronize in vitro nuclear and cytoplasmic maturation using CNP as an OMI.^15^ In the CAPA‐IVM approach, cumulus‐oocyte complexes isolated from small antral follicles are pre‐matured in a medium containing CNP, estradiol, insulin, and FSH, before underdoing IVM. A previous clinical study demonstrated improvement in yielding high‐quality embryos.^63^ However, the effect of CNP is likely inadequate due to low expression levels of NPR2 in cumulus cells derived from small antral follicles.^22^ Based on our present findings, the protocol could be optimized by introducing ODFs in the pre‐maturation medium. This modification may enhance the effectiveness of the CAPA‐IVM approach and potentially improve clinical outcomes for patients undergoing IVM treatment.

FSH is commonly used in COS to promote the simultaneous development of multiple antral follicles in the ovary for oocyte retrieval in IVF programs. This study aimed to identify a pharmacological agent capable of inducing early follicle development during the FSH‐independent phase. Several substances have been identified as potential inducers of early follicle development, including activin,^64^ insulin‐like growth factor,^65^ and r‐spondin 2.^66^ However, these agents have yet to be formally adopted in clinical practice. Patients with POI and DOR have a limited number of residual primordial and developing follicles, resulting in few antral follicles at the initiation of COS. Consequently, COS using FSH is often ineffective, yielding either no oocyte or only a limited number. Therefore, developing methods to induce early‐stage follicle growth and increase the number of follicles responsive to FSH is crucial for creating effective COS protocols for patients with POI and DOR. The findings of our study suggest that the concurrent administration of CNP and E2 prior to COS in patients with POI and DOR could potentially serve as an effective protocol to promote the growth of early‐stage follicles. This approach may ultimately lead to the development of an optimal protocol for these patients.

Recently, vosolitide, a human CNP analog, has been approved in Japan for the treatment of patients with chondrodysplasia and is being applied clinically.^67^ It is anticipated that vosolitide will induce the proliferation of human granulosa cells and promote early‐stage follicle development, and potentially represent a promising treatment option for patients with POI and DOR. Nevertheless, further studies are required before vosortide can be considered a candidate for clinical application in infertility treatment due to its vasodilatory properties, which result in anti‐hypertensive effects.^27^

## CONFLICT OF INTEREST STATEMENT

Kazuhiro Kawamura and Yorino Sato declare no conflict of interest.

## ETHICS STATEMENT

This article does not contain any study with human participants that has been performed by any of the authors.
